# Antibiotic resistance genes prevalence prediction and interpretation in beaches affected by urban wastewater discharge

**DOI:** 10.1016/j.onehlt.2023.100642

**Published:** 2023-10-11

**Authors:** Qandeel Zahra, Jawaria Gul, Ali Raza Shah, Muhammad Yasir, Asad Mustafa Karim

**Affiliations:** a Azra Naheed Medical College, Lahore 54000, Punjab, Pakistan; b Al-Nafees Medical College & Hospital, Islamabad 44000, Pakistan; c Special Infectious Agents Unit, King Fahd Medical Research Center, King Abdulaziz University, Jeddah 21589, Saudi Arabia; d Department of Oriental Medicine and Biotechnology, College of Life Sciences, Kyung Hee University, Yongin-si 17104, South Korea; e Department of Medical Laboratory Sciences, Faculty of Applied Medical Sciences, King Abdulaziz University, Jeddah 21589, Saudi Arabia

**Keywords:** Antibiotic resistance genes, Environment, Beach waters, Machine learning, Explainable artificial intelligence

## Abstract

**Background:**

The annual death toll of over 1.2 million worldwide is attributed to infections caused by resistant bacteria, driven by the significant impact of antibiotic misuse and overuse in spreading these bacteria and their associated antibiotic resistance genes (ARGs). While limited data suggest the presence of ARGs in beach environments, efficient prediction tools are needed for monitoring and detecting ARGs to ensure public health safety. This study aims to develop interpretable machine learning methods for predicting ARGs in beach waters, addressing the challenge of black-box models and enhancing our understanding of their internal mechanisms.

**Methods:**

In this study, we systematically collected beach water samples and subsequently isolated bacteria from these samples using various differential and selective media supplemented with different antibiotics. Resistance profiles of bacteria were determined by using Kirby-Bauer disk diffusion method. Further, ARGs were enumerated by using the quantitative polymerase chain reaction (qPCR) to detect and quantify ARGs. The obtained qPCR data and hydro-meteorological were used to create an ML model with high prediction performance and we further used two explainable artificial intelligence (xAI) model-agnostic interpretation methods to describe the internal behavior of ML model.

**Results:**

Using qPCR, we detected *bla*_CTX−M_, *bla*_NDM_, *bla*_CMY_, *bla*_OXA_, *bla*_tetX_, *bla*_sul1_, and *bla*_aac(6′-Ib-cr)_ in the beach waters. Further, we developed ML prediction models for *bla*_aac(6′-Ib-cr)_, *bla*_sul1_, and *bla*_tetX_ using the hydro-metrological and qPCR-derived data and the models demonstrated strong performance, with R2 values of 0.957, 0.997, and 0.976, respectively.

**Conclusions:**

Our findings show that environmental factors, such as water temperature, precipitation, and tide, are among the important predictors of the abundance of resistance genes at beaches.

## Highlights

• Antibiotic resistance is most serious public health threat.
• ARGs were detected at recreational beaches by qPCR.
• ML models were developed to predict ARGs on beaches.
• Explainable Artificial Intelligence was used to explain ML models.
• Tide, rain, and temperature affected ARGs abundance at beaches.

## 1 Introduction

The excessive and inappropriate use of antibiotics in medical and agricultural practices has led to the release of these drugs into the environment, significantly contributing to the emergence and dissemination of resistant bacteria and antibiotic resistance genes (ARGs). Consequently, the effectiveness of numerous essential antibiotics has been compromised, resulting in a significant number of deaths and extended hospital stays [1]. Although most studies on resistance spread have focused on clinical settings [2], there is increasing recognition of antimicrobial resistance (AMR) as a “One Health” issue, highlighting the interconnectedness of human health, animal health, and the natural world [2,3]. While a majority of studies on the spread of antibiotic resistance have focused on clinical settings [2], there is a growing acknowledgment of antimicrobial resistance (AMR) as a “One Health” issue, emphasizing the interdependence of human health, animal health, and the environment [2,3]. AMR can emerge and spread throughout different ecosystems, affecting both humans and animals. This is often facilitated by a variety of factors, such as human movement and travel, transportation of livestock and food products, and insufficient waste disposal practices [2,3,4].

The Earth's surface is composed of 71% water, with various water bodies connected, providing a potential means for bacteria to spread antibiotic resistance (AbR). Human sewage waste is a significant contributor to the dissemination of resistant bacteria in the ecosystem, via human waste, urine, and various sewage wastes. Furthermore, bacteria, genetic materials (integrons, plasmids, and transposons) can infiltrate coastal recreational areas through runoff from landfills and sewage treatment facilities [5]. This scenario creates the potential for ARG transmission from animal and human bacteria to marine bacterial populations or exposed humans. Therefore, recreational beaches are particularly vulnerable to ARG contamination from multiple channels, including animal feed industry, sewage treatment plants, and storm-water runoff. Studies have shown that bodyboarders, surfers, and beachgoers, have a higher probability of carrying resistant bacteria in their gastrointestinal tract than non-surfers [2,4]. Therefore, it is crucial to monitor the spread of ARGs in recreational beaches to safeguard the health and well-being of the public engaged in beach activities.

To investigate the presence of ARGs in water ecosystems and the success of stewardship in reducing their concentration, researchers have developed various molecular methods such as quantitative polymerase chain reaction (qPCR), metagenomics, and traditional PCR. However, conventional beach water quality monitoring programs typically focus on a limited set of indicator bacteria, often overlooking surveillance of resistant bacteria and ARGs. These bacteria carrying ARGs possess the potential to integrate into natural reservoirs. The dissemination of these genes into coastal areas can have significant implications for public health and food security. Furthermore, there are several limitations to the methods used to monitor ARG concentration. Traditional microbiology techniques are time-consuming, while metagenomics and qPCR are faster and trustworthy methods in identifying and quantifying ARGs. However, these methods can be costly and can produce nonspecific amplification products, limiting their usefulness for routine monitoring [4]. Therefore, it is necessary to develop and use predictive tools for highly effective and straightforward AMR surveillance to ensure safety of the public.

The use of machine learning (ML) has become increasingly popular in the fields of environmental management and healthcare to aid in decision-making processes that have an impact on human health. Previous studies have utilized a variety of ML algorithms, such as Support Vector Machine, Random Forest, and Artificial Neural Networks, to predict ARGs in different environments, including soil, food, and natural aquatic habitats [[6], [7], [8], [9]]. However, the absence of clarity and accountability in these models can lead to unforeseen consequences when they are deployed for critical applications [4]. Therefore, it is important to address these issues and ensure that ML models are reliable and trustworthy before implementing them in sensitive contexts.

Machine learning (ML) has been employed in various fields, including healthcare, water quality, and environmental management, to predict and forecast problems. For instance, authors developed models to predict ARGs occurrence in the beaches of South Korea (Busan) [10]. Nevertheless, ML models are frequently perceived as opaque or “black boxes”, signifying that their internal workings are not transparent or interpretable, and humans are unable to comprehend their inner logic [[11], [12], [13], [14]]. This can lead to serious consequences when deploying them for sensitive or important applications. Therefore, it is essential to create interpretable and trustworthy ML models that can offer explanations for their predictions, especially in high-stake decision-making contexts [12,15]. Limited research has been conducted on the application of interpretive approaches to describe the predictions of black-box ML models in the context of ARGs prediction. In this study, we used two state-of-the-art interpretation techniques to interpret an ML model's predictions for ARGs occurrence in two locations' coastal waters: South Korea (Busan) and Pakistan (Karachi, and Balochistan). By utilizing hydro-meteorological time-series data, we were able to make accurate predictions, which can enhance the credibility and adoption of ML models.

## 2 Methods

### 2.1 Sites for sampling and sample collection

The research was conducted in two regions, Karachi and Hub, located in Sindh and Balochistan, Pakistan, respectively. The latitude of the study area extends between 24.8096° N and 25.1186° N, while its longitude spans from 66.7267° E to 67.0115° E. The selection of Karachi's beaches as a representative sample was based on the city's high population density, significant tourism, and environmental impact, which are common features of many coastal cities. These beaches receive discharge of treated wastewater from various sources, including households, local industries, and hospitals within the city. Karachi is the world's twelfth-largest city and the most populous city in Pakistan. The width of Clifton Beach varies between 25 and 110 m, and its total area is 82,000 m^2^. Seawater samples were collected below 1 m of the surface during May to July 2020 and during rainy events. The researchers collected 156 samples from Pakistan and stored them in containers at 4 °C in the dark for further analysis. Further, Jang et al.'s previous study provided 218 samples that were included in our ML analysis [10].

### 2.2 Environmental elements and bacterial growth

The development of the models in this study involved the use of six categories, containing three sets of climatic parameters (air pressure, wind speed, and total rainfall for 1 h and 4 h) and three sets of water variables (salinity, tides, and water temperature). To train the model, we collected a total of 536 data points, which were recorded at 30-min intervals, with variations in the number of data points for each event. To account for the significant influence of antecedent rainfall on bacteria occurrence in beach environments, the model incorporated rainfall data for 1 h and 4 h as input variables, as previously established [10]. The bacterial culture was conducted using MacConkey agar plates, a selective and differential media that can differentiate between lactose- and non-lactose fermenting pathogens based on colony colour. A sterile loop was used to streak the bacterial culture onto the agar plates, which were further incubated upside down at 37 °C for 18–24 h, according to previous reports [16,17]. Distinct bacterial colonies were picked and streaked onto fresh plates to ensure pure isolates. Isolated bacteria were identified by 16 s ribotyping.

### 2.3 Antibiotic susceptibility profiles

In order to assess the antibiotic susceptibility of the isolates, 12 different antibiotics from various classes were used in this study. The determination of susceptibility profiles was performed using the Kirby-Bauer disk diffusion method [18], and the interpretation of the zone diameters was based on the 2019 CLSI guidelines [19]. In brief, the procedure involved preparing a 0.5 McFarland standard inoculum from overnight cultures, followed by incubating the inoculated Mueller-Hinton agar plates. All plates were then placed in the incubator and left for a 16-h incubation period. Subsequently, Antibiotic discs with known concentration of antimicrobials were placed on the agar surface using sterile forceps and gently pressed down to ensure optimal contact and incubated for 24 h at 37 °C. Antibiotic discs with known concentration of antimicrobials were placed on the plates and subsequently incubated for 24 h at 37 °C. Isolates were tested for following classes of antimicrobials; aminoglycosides (amikacin (30 μg) and kanamycin (30 μg)), penicillins (ampicillin (10 μg) and piperacillin (30 μg)) cephalosporins (cefotaxime (30 μg), ceftazidime (30 μg), and cephalothin (30 μg)), fluoroquinolones (ciprofloxacin (5 μg)), polymyxins (colistin (10 μg)), carbapenems (imipenem (10 μg)), tetracyclines (tetracycline (30 μg)), and sulfonamides (sulfamethoxazole (5 μg)).

### 2.4 DNA extraction

Every sample was membrane-filtered in a vacuum using 0.45 m nylon membrane screens before the bacteria were dropped in 10 mL of saline within 6 h. The concentrated microbial samples were kept after centrifugation of this suspension for deoxyribonucleic acid (DNA) extraction. Using a QIAGEN DNA Extraction Kit (Maryland, USA), total DNA was extracted from each sample in accordance with the manufacturer's recommended procedures. The collected DNA was preserved for future analysis at a temperature of −20 °C.

### 2.5 ARGs detection by PCR

To enumerate ARGs by qPCR, a SolGent 2 x Real-Time Smart Mix (20 μL) was prepared with specific forward (0.4 μL) and reverse (0.4 μL) primers, 8.0 μL of PCR-grade water, and 1 μL of DNA template. The master mix was then subjected to 39 cycles of amplification using the following three-step protocol: denaturation at 95 °C for 3 min, optimal annealing temperature at 66.0 °C for *aac*(*6’-Ib-cr*), 56.0 °C for *sul*1, and 61.0 °C for *tet*X for 3 min, and extension at 72 °C for 10 s [10].

### 2.6 Machine learning models (English correction)

Gradient boosting decision tree (GBDT) is a widely used ML approach in environmental studies. The weak learner's decision tree ensemble serves as the foundation for the GBDT prediction model [7,20,21]. To boost robustness and prevent overfitting, every weak classifier was trained using various combinations of training data. In this work, we used CatBoost, a quick and effective implementation of Yandex's 2017 GBDT algorithm with outstanding prediction performance [11,15,22]. Using a random splitting strategy, the initial data were separated into a non-overlapping training set (80%) and a testing set (20%), yielding 299 samples in training with 75 samples to test. Using training data, the network optimization was carried out. We employed mean square error (MSE) among real and simulated ARGs as a gradient descent during training. In addition, we assessed and compared model performance using Nasch-Sutcliff Efficiency (NSE), co-efficient of determination (R2), and root mean squared logarithmic error (RMSLE) and others.

To properly evaluate the performance of models, the use of multiple independent performance metrics capturing different properties of the prediction is essential. Naser and Alavi (2021) have recommended this approach, along with Ribeiro et al. (2016) [12,15]. In this study, we have selected R2, Percentage BIAS (PBIAS), NSE, RMSE, correlation coefficient, and mean squared logarithmic error (MSLE) as performance measures. We have included these metrics as they encapsulate various aspects of model effectiveness and are influenced by outliers to varying extents. The choice of RMSLE was made due to its relative insensitivity to outliers. Among them, R2 is an important statistical measure that is widely used to estimate the effectiveness of prediction models, such as regression and ML models. An R2 value of 1 indicates a perfect fit, meaning that all of the variance in the dependent variable can be explained by the independent variables used in the model. An R2 value closer to 0 indicates a weaker fit, meaning that a smaller percentage of the change in the dependent factor can be described by the independent variables. The formulas to calculate the performance metrics are given below;

$$\mathrm{MSE}=\frac{\left[\sum_{i=1}^{n}{\left(o_{i}-p_{i}\right)}^{2}\right]}{n}\#$$

$$\begin{array}{c} \mathrm{NSE}=1-\frac{\sum {\left(o_{i}-p_{i}\right)}^{2}\;}{\sum {\left(o_{i}-\bar{o}\right)}^{2}\;}\# \end{array}$$

$$\mathrm{RMSLE}=\sqrt{\frac{1}{n}\sum_{i=1}^{n}{\left(\log\left(p_{i}+1\right)-\log\left(o_{i}+1\right)\right)}^{2}}$$

$$R2\;\mathrm{score}\;\left(p^{\prime },o\right)=1–\frac{\sum_{n=1}^{N}{\left(p_{n}^{\prime }-{\mathrm{xo}}_{n}\right)}^{2}}{\sum_{n=1}^{N}{\left(o_{n}-\bar{o}\right)}^{2}}$$

$$\mathrm{BIAS}=\frac{\sum_{i=1}^{n}o_{i}-p_{i}}{\sum_{i=1}^{n}o_{i}}$$

Where o_i_ and p_i_ are the observed and predicted values, respectively, and n indicates the number of samples in the training and test dataset.

$$\mathrm{ALE}=\mathrm{ALEi}\left(x\right)=N1\sum j=1\;N\left[\mathrm{Fi}\left(x,j\right)-\mathrm{Fi}\left(x,j-1\right)\right].$$

*ALEi* (*x*) is the *Average Local Effect* for the i-th feature at the input point x.

*Fi* (*x, j*) is the model's prediction when feature *i* is set to its *j*-th order statistic (sorted value of feature *i*).

*N* is the number of unique values for feature *i*.

### 2.7 Interpretation

Through the analysis of tree splits and calculating the average variation in the CatBoost model's predictions based on changes in an input feature, it is possible to interpret the CatBoost model. [12,23]. For understanding and explaining the behavior of the trained CatBoost model in relation to the input features, we used two model-independent interpretation approaches [24]. These include accumulated local effects (ALE) and local interpretable model agnostic explanation (LIME). Accumulated local effects (ALE) plots are an alternative to PD plots, which do not require this unreliable extrapolation between correlated input features [25]. Previous methods mainly focus on understanding the overall behavior of the ML model. Conversely, the LIME method aims to investigate the local behavior of the model. LIME employs a surrogate interpretable linear regression model to explain the prediction of a complex ML model for an individual sample [26]. To train the linear regression model, new input data points are generated within the local neighborhood of the original sample. However, LIME suffers from in-stability and an unresolved definition of neighborhood, giving us inconsistent interpretation results from LIME on multiple iterations [27,28]. The complete machine learning pipeline from data-preprocessing, to building and training of models, predictions, xAI, and analysis of results was performed using AI4Water which is a python based framework for performing aforementioned tasks.

### 2.8 Code availability

The GitHub repository (link: https://github.com/Asadmalic/arg_prediction_ml) includes the code necessary to replicate the results presented in this article. All the figures shown in this study are fully executable and reproducible.

## 3 Results and discussion

### 3.1 Sample collection

A total of 156 samples were collected from Karachi, and an additional 218 samples were included from a previous study conducted in Korea [10]. The MacConkey agar was used as the culture medium to cultivate the major human pathogens. The samples were found to contain common Gram-negative pathogens, including *E. coli*, *Klebsiella pneumoniae*, *Acinetobacter baumannii*, and *Pseudomonas aeruginosa*, as reported in previous studies [7,29].

### 3.2 ARGs detected in beach water samples

The objective of our study was to identify the prevalence of most frequently occurring ARGs in beach environments. We identified *bla*_aac(6′-Ib-cr)_, *bla*_sul1_, and *bla*_tetX_ as the most commonly occurring ARGs. Therefore, we decided to focus on these three ARGs for the development of our machine learning models, aiming to maximize the number of input variables. On the other hand, we found that *bla*_NDM_, *bla*_OXA_, *bla*_CMY_, and *bla*_CTX−M_ were not detected in all the samples. Therefore, we opted not to incorporate them into ML model development.

We conducted a comprehensive analysis on the collected samples to detect the presence of 12 distinct ARGs, which have the potential to confer resistance against a range of antibiotics. The susceptibility profiles of the isolates against each antibiotic is given in Table 1. We used the primers for four class of β-lactamases and for other ARGs. The detected genes belong to class A β-lactamase (*bla*_CTX−M_), class B β-lactamase (*bla*_NDM_), class C β-lactamase (*bla*_CMY_), class D β-lactamase (*bla*_OXA_), tetracycline resistance (*bla*_tetX_), sulfonamide resistance (*bla*_sul1_), and aminoglycosides resistance (*bla*_aac(6′-Ib-cr)_ [30,31]. Further, we detected *Pseudomonas aeruginosa, E. coli*, *Acinetobacter baumannii, and Klebsiella pneumoniae* in all the collected samples from the beaches. We detected *bla*_tetX_, *bla*_sul1_ and *bla*_aac(6′-Ib-cr)_ in all the isolates, whereas *bla*_NDM_ and *bla*_OXA_ genes were predominant in all the isolates (72%) followed by *bla*_CMY_ (48%) and *bla*_CTX−M_ (32%). These results suggest that beach environments could be a possible reservoir for antibiotic resistance [4,10]. In another study, authors analyzed the prevalence and diversity of β-lactamases, including *bla*_CTX−M_, *bla*_NDM_, and *bla*_OXA_ among *Escherichia coli* isolates from urban wastewater treatment plants and agricultural fields [32,33]. Similarly, a similar study was published on the detection of ARGs on beaches using qPCR [10], in which the authors employed ML models and explainable artificial intelligence to predict the ARGs in beaches [10]. Samples taken from the Karachi beaches showed the greatest concentration of ARGs among all the samples examined. In 2018, a comprehensive global data was published on the usage of antibiotic over a period of 16 years (2000–2015) in in 76 different countries of the world [33]. According to the results, there was a 65% increase in antibiotic consumption in the world. It was reported in low- and middle-income countries, there was substantial increase in the usage of antibiotics. According to recent reports, there has been a significant increase in the use of antibiotics in low- and middle-income countries, which has contributed to the development of antibiotic resistance. A recent study found that overall antibiotic usage amplified by 65% between 2000 and 2015, with the majority of this increase occurring in LMICs [33]. The rise in antibiotic consumption within LMICs can be attributed to factors such as population growth, enhanced access to healthcare services, and increased utilization of antibiotics for livestock and aquaculture purposes. This trend is particularly concerning given the high burden of infectious diseases in LMICs and the limited resources available to combat antibiotic resistance [33]. Based on the results, it was observed that there is an increasing trend in antibiotic usage in Pakistan (65%), making it the third-largest country in terms of high antibiotic consumption globally, following India (103% increase) and China (79% increase) [33]. The observed increase in the usage of last-resort antibiotic classes is a cause for significant concern, as it could lead to the emergence of highly drug-resistant bacteria and genes, as indicated in our study. This trend in antibiotic consumption was consistent across low-, middle-, and high-income countries, including the use of polymyxins, oxazolidinones, carbapenems, and glycylcyclins. Particularly noteworthy is the rise in the usage of carbapenems and polymyxins, notably colistin, in low- and middle-income nations during the study period. This rise could be attributed to antibiotic-induced selection pressure on bacteria, raising alarm about the presence of ARGs in beach environments.

**Table 1:** Antibiotic resistance profile of isolates against all tested antibiotics.

	Antibiotic	Resistance profile (%)
1	Ciprofloxacin	95%
2	Chloramphenicol	50%
3	Nalidixic acid	85%
4	Ampicillin	100%
5	Ceftazidime	82%
6	Cefotaxime	100%
7	Cefepime	80%
8	Aztreonam	78%
9	Meropenem	75%
10	Gentamicin	63%
11	Fosfomycin	8%
12	Sulfamethoxazole-trimethoprim	95%

Li et al. investigated the microbial community ARGs in severely contaminated beach sediments from a Chinese beach, using metagenomic sequencing methods [34]. They identified a high abundance of ARGs, including those conferring resistance to multiple classes of antibiotics. In a related study, Czekalski and colleagues investigated the occurrence and distribution of ARGs in Lake Geneva, Switzerland [35]. Their results indicated a wide distribution of ARGs across the lake, particularly in areas impacted by wastewater discharge [35]. Similar to our study, Zhu et al., investigated the occurrence of ARGs in seawater and beach sand in Hawaii. They found that both environments contained a diverse array of ARGs, including those associated with resistance to antibiotics commonly used in human and veterinary medicine [36].

In the current study, carbapenemases and multiple ARGs were found to be co-prevalent with ARGs. Thapaliya and colleagues conducted research that revealed the prevalence and characterization of *Staphylococcus aureus* and Methicillin-Resistant *S. aureus* strains on public recreational beaches [37]. These findings suggest that human activity could contribute to the pathogenic contamination of beach water and sand [37]. The *bla*_NDM_-type ESBLs have been identified globally as the primary source of resistance to third-generation cephalosporins in bacteria isolated from various samples, including clinical and environmental samples. Multidrug resistance-producing bacteria colonization rates in humans are estimated to be around 14% globally, and resistance rates may be even higher, up to 22%, in Sub-Saharan Africa and Asia [38]. In this study, we reported different ARGs from different beta-lactamases classes from the environment (beach) isolated bacteria. This revealed relatively higher ARGs burden in the beach environment as compared to the previous published studies. In a similar study, Ding et al., reported that the effluent from the wastewater treatment plant contained a high abundance of ARGs, including those conferring resistance to carbapenems, a critically important class of antibiotics [39]. The receiving surface water also had a significant abundance of ARGs, indicating that the effluent from the wastewater treatment plant was contributing to the spread of antibiotic resistance in the environment [40]. According to a recent published report on the wastewater, 35.29% *bla*_TEM_ ARGs were reported and about 64% *bla*_CTX-M_ were reported in the study [41]. Our study detected a higher number of ARGs, which is consistent with previous research that found similar patterns [4,10,41]. Specifically, *bla*_NDM_ was the most prevalent gene, followed by *bla*_OXA_ and *bla*_CMY_. Differences in carbapenemase patterns among bacteria from different geographical regions may be attributed to differences in prevalent sequence types (STs) with varying resistance mechanisms [29].

### 3.3 Prediction performance

Recreational beaches were analyzed for the presence of ARGs using qPCR in the current study. ML models were developed with high prediction performance to predict ARGs. The evaluation frameworks in different fields depend significantly on performance metrics. Different studies employ the R2, NSE, RMSE, RMSLE, PBIAS, correlation coefficient and MSLE [10,42]. The evaluation of regression-based ML models relies on several performance metrics, among which the R2 score is an essential one. The R2 score is a significant measure that assesses the performance of the regression-based ML model [10]. We first trained the model using the training set and subsequently applied the trained model to the test set. We saved the results of the regression analysis, which were measured through a variety of metrics, including NSE, R2, RMSE, PBIAS, RMSLE, correlation coefficient, and MSLE. In our study, the R2 value was very high as compared to other studies, meaning that the prediction is highly correct [10]. Other performance metrics are reported in the Table 2. A study similar to ours was conducted by Jang et al., where they investigated the presence of ARGs on recreational beaches. However, the R2 value reported by the authors was comparatively lower [10]. The study conducted by the researchers reported R2 values for both training and testing. The R2 values for training varied between 0.50 and 0.87, while for testing, the R2 values ranged from 0.32 to 0.65. However, in the present study, R2 value was higher for training and testing. Hence this indicates that the greater neural network training accuracy compared to that of testing indicates that the neural network was over fitted [10].

**Table 2:** Performance metrics of CatBoost model in training and testing.

	R2	NSE	RMSE	rmsle	PBIAS	corr_coeff”	msle
*bla*_*aac(6′-Ib-cr)*_							
Test	0.9579	0.9569	1,858,252.199	2.664	−9.592	0.9787	7.097
Train	0.9999	0.9999	143.864	0.040	−0.0001	0.9999	0.001
Validation	0.9579	0.9567	1,858,252.199	2.664	−9.592	0.9787	7.097
*bla*_*sul*1_							
Test	0.9974	0.9972	3,674,296.941	1.388	−2.656	0.9987	1.928
Train	0.9997	0.9818	16,433,519.130	0.089	−9.050	0.9998	0.008
Validation	0.9974	0.9972	3,674,296.941	1.388	−2.656	0.9987	1.928
*bla*_tetX_							
Test	0.9761	0.9723	2,295,469.169	2.545	−27.528	0.9879	6.478
Train	0.9998	0.9996	374,349.341	0.0318	−0.969	0.9999	0.001
Validation	0.9761	0.9723	2,295,469.169	2.545	−27.528	0.9879	6.478

A comparison of the performance metrics of the CatBoost model for training, testing, and validation of the three detected genes is presented in Table 2**.** The results indicate that *bla*_sul1_ was overestimated during September 2019 in Busan, while *bla*_aac(6′-Ib-cr)_ was underestimated during August 2019 in Busan. The XGBoost model's individual performance metrics for all ARGs are also reported in Table 2, including R2, NSE, RMSE, RMSLE, PBIAS, correlation coefficient, and MSLE.

The CatBoost model was used for the prediction of *bla*_sul1_ (CatB_*bla*_sul1_), *bla*_tetX_ (CatB_*bla*_tetX_), and *bla*_aac(6′-Ib-cr)_ (CatB_*bla*_aac(6′-Ib-cr)_). The optimization of the hyperparameters of the models was performed using the Bayesian optimization method. The results indicate that a higher value of the learning rate led to improved R2 and NSE values, as shown in the Table 2. The performance metrics of the CatBoost model were evaluated during both training and testing, and the results are presented in the Table 2. R2, NSE, and RMSLE were monitored for all ARGs. R2 was 0.957, 0.99 and 0.976 for *bla*_aac(6′-Ib-cr)_, *bla*_sul1_ and *bla*_tetX_ respectively, for test data (Table 2). The test score in terms of NSE was 0.956, 0.997, and 0.972 for all three ARGs. Although the R2 and NSE were highest among all ARGs, RMSLE was the lowest for blasul1 (Table 2). Previous studies have reported similar results to our findings, where the R2 values for the prediction of ARGs were 0.65, 0.52, and 0.67 for *bla*_aac(6′-Ib-cr)_, *bla*_sul1_, and *bla*_tetX_, respectively [2,4,6,10]. However, the R2 values were greater than 0.95 in the present study, indicating higher prediction performance of our model.

### 3.4 Accumulated local effects (ALE)

**Fig. 1:**
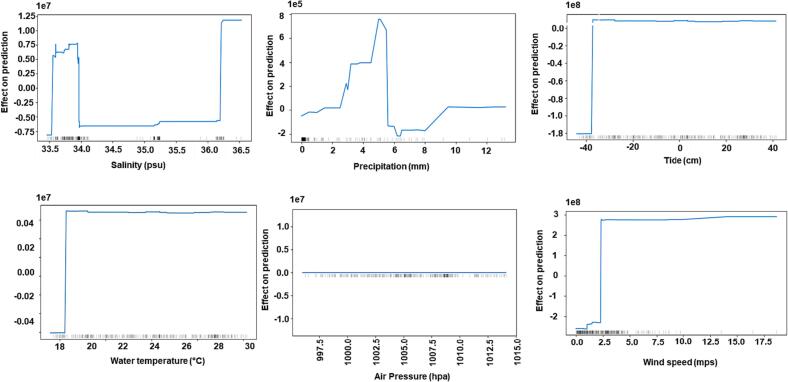
Accumulated local effect graphs showing effect of each input feature on prediction of CatBoost_ *bla*_aac_(*6′-Ib-cr*). The distribution of an input feature in the training data is shown by gray bars.

**Fig. 2:**
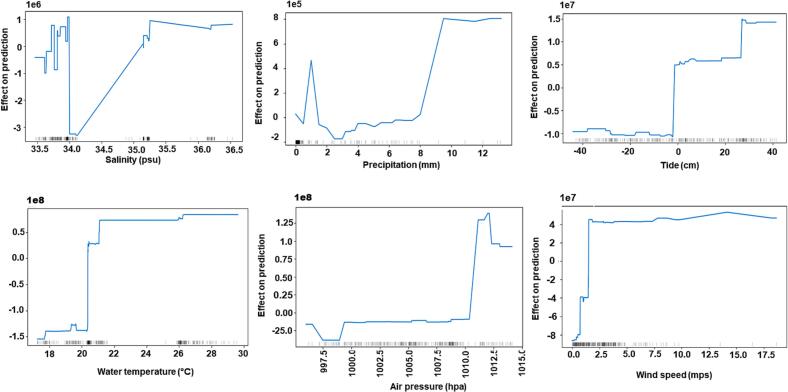
Accumulated local effect graphs showing effect of each input feature on prediction of CatBoost_ *bla*sul*1*. The distribution of an input feature in the training data is shown by gray bars.

**Fig. 3:**
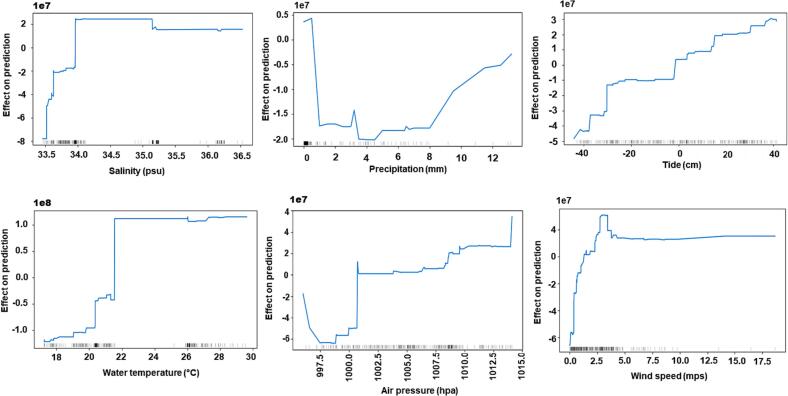
Accumulated local effect graphs showing effect of each input feature on prediction of CatBoost_ *bla*tet*X*. The distribution of an input fea-ture in the training data is shown by gray bars.

Explainable Artificial Intelligence explained black-box models' behavior for ARGs. We used the ALE approach to extract the importance of each input feature for the ML model. ALE illustrates how changes in features influence the output of a function. The results of ALE for CatB_ *bla*_aac(6′-Ib-cr)_ (Fig. 1), CatB_*bla*_sul1_ (Fig. 2), and CatB_*bla*_tetX_ (Fig. 3) models are shown in Figures, respectively. These figures show that the tide's impact on model performance differs significantly from that of other plots. The ALE plots showed that the prediction of all models increases as we increase the tide's value [4]. The prediction for CatB_ *bla*_aac(6′-Ib-cr)_ increases as the tide increases above −38 cm. The prediction of ARGs decreases for CatB_*bla*_sul1_ and CatB_*bla*_tetX_ when tide values fall below zero. The model's prediction for precipitation increases as its value increases above 8 cm.

In contrast to other plots, ALE plots showed that the model's behavior in regions with little to no training data is linear. ALE plots (Fig. 1, Fig. 2, Fig. 3) visualize the distribution of an input feature in the training data using gray bars. This is evident from regions of salinity between 34.2 and 35 practical salinity unit (psu) and for a water temperature between regions of 22 and 26 °C. ALE performs a linear interpolation in the model's behavior in regions with no training data. We also observed that all three models show constant behavior with an increase in wind speed above 10 mps. This can also be attributed to the fact that all models were trained with data in which very few samples were available with wind speed above 10 mps.

### 3.5 Local interpretable model explanations (LIME)

**Fig. 4:**
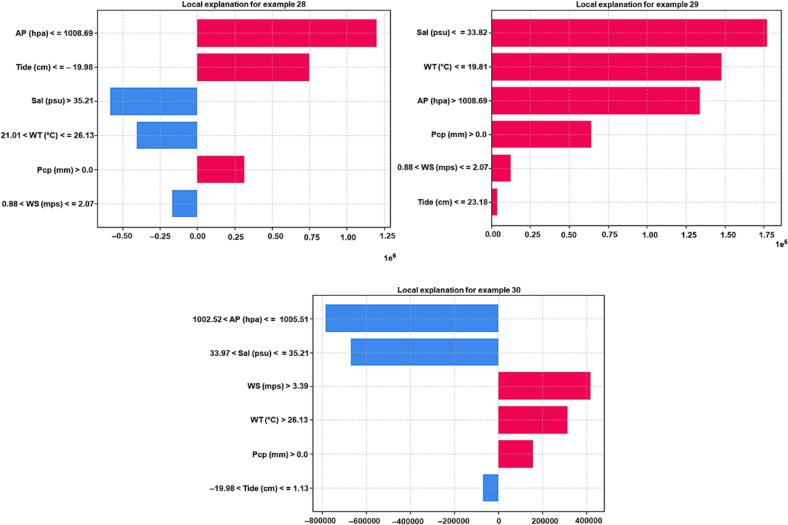
Bar graphs show local feature importance of each input feature for sample number 28, 29, and 30 using LIME method for CatBoost_ *bla*aac(*6′-Ib-cr*) model. Blue bars represent decrease in ARG while red bars represent increase in ARG by CatBoost_ *bla*aac(*6′-Ib-cr*) model. WT: water temperature; WS: wind speed; AP: air pressure; Pcp: precipitation; Sal: salinity. (For interpretation of the references to colour in this figure legend, the reader is referred to the web version of this article.)

**Fig. 5:**
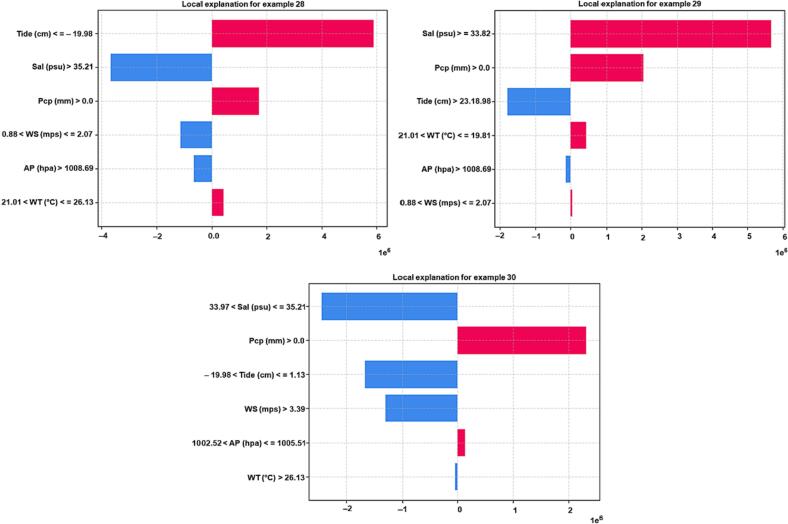
Bar graphs show local feature importance of each input feature for sample number 28, 29, and 30 using LIME method for CatBoost_ blasul1 model. Blue bars represent decrease in ARG while red bars represent increase in ARG by CatBoost_ *bla*sul1 model. WT: water temperature; WS: wind speed; AP: air pressure; Pcp: precipitation; Sal: salinity. (For interpretation of the references to colour in this figure legend, the reader is referred to the web version of this article.)

**Fig. 6:**
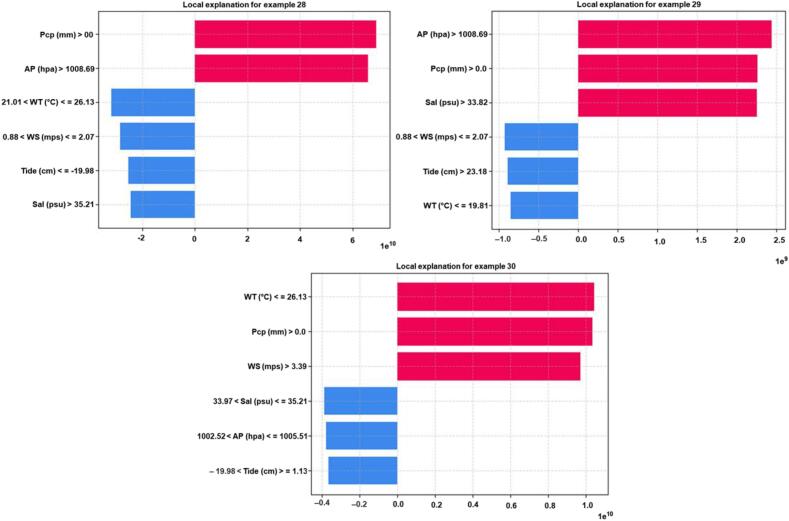
Bar graphs show local feature importance of each input feature for sample number 28, 29, and 30 using LIME method for CatBoost_ *bla*tetX model. Blue bars represent decrease in ARG while red bars represent increase in ARG by CatBoost_ *bla*tetX model. WT: water temperature; WS: wind speed; AP: air pressure; Pcp: precipitation; Sal: salinity. (For interpretation of the references to colour in this figure legend, the reader is referred to the web version of this article.)

To explain the local behavior of the CatBoost model using LIME, we focused on a high peak event from the test data. This event happened for sample number 29 when the ARG prediction from CatB_*bla*_aac(6′-Ib-cr)_ (Fig. 4), CatB_*bla*_sul1_ (Fig. 5) and CatB_*bla*_tetX_ (Fig. 6) reached 7.4e^7^, 6e^8^, and 1.2e^8^ copies per milliliter (coppml), respectively.

Figure shows that the high ARG prediction for sample number 29 corresponds with an increase in precipitation, tide, and air pressure and a decrease in salinity and water temperature. The explanations from LIME for the three CatBoost models for samples 28 to 30 are shown in Fig. 4. When the *bla*_aac(6′-Ib-cr)_ prediction is low for samples 28 and 30, some features contribute to a decrease in the ARG prediction while others cause an in-crease in ARG prediction. However, for sample number 29, all input features increase the model's prediction [10].

The salinity was found to have the greatest impact on the ARGs, according to Fig. 4, while the tide was found to have the least impact. It is worth noting that, for sample number 29, the model's prediction increased when salinity values were lower than 33.8 psu and water temperature values were below 19.8 °C [10]. For CatB_*bla*_sul1_ and CatB_*bla*_tetX_ models, salinity and precipitation were key input features that caused an increase in model prediction [4,10]. However, the causes of prediction were different from each other. The precipitation caused an increase in ARG prediction only when it was greater than 0.0 mm. On the other hand, salinity values below 33.8 psu caused an increase in model prediction. We observed that water temperature, precipitation, and tide greatly affected ARGs abundance as demonstrated by the results presented in the study. Furthermore, the post-processing methods introduced here can serve as a valuable tool for debugging machine learning models.

## 4 Limitations

The present study used small number of data points, which is relatively low compared to the ideal sample size. The collection, analysis, and quantification of ARGs from water samples is a time-consuming and costly process, which can limit the number of data points that can be collected. It should be noted that a previous study by Jang et al. (2021) developed their ML models using only 218 samples [10]. Our study aimed to supplement Jang's data in order to create a more generalizable model and explain its behavior. However, it is important to acknowledge that the number of data points in our study is still limited. To develop a more comprehensive and robust ML model, it is recommended to collect additional samples beyond those used in this study.

Another limitation of this study is that it did not account for all the hydro-climatic parameters, which could influence the occurrence and distribution of ARGs in the beach environment. Future research should examine the connections between hydro-climatic conditions and the prevalence and variety of ARGs in beach sediment and water to gain a better understanding of their contribution to the dissemination of antibiotic resistance.

In our study, we comprehensively considered key hydro-meteorological factors (such as water temperature, salinity, precipitation (rainfall), air pressure, tide, and wind speed) that are known to influence the dissemination of antibiotic-resistant bacteria and ARGs. However, we acknowledge that our data collection did not encompass certain other critical hydro-meteorological factors that can affect bacterial growth and ARG prevalence. Notably, we did not collect data on pH, humidity, and exposure to UV radiation/sunlight, all of which are recognized for their substantial roles in bacterial growth and survival. Future research should consider integrating these parameters to provide a more comprehensive understanding of the hydro-meteorological influences on antibiotic resistance dynamics.

## 5 Conclusions

The results of our research indicate that water temperature, precipitation, and tide are the primary factors driving the abundance of ARGs in recreational beaches. We developed a machine learning model that utilized hydro-meteorological and qPCR data from South Korea and Pakistan, achieving high prediction performance. Furthermore, we utilized two explainable artificial intelligence model-agnostic explanation methods to explain the behavior of our model. These methods not only provided insight into the behavior of the model under new conditions but also served as a useful tool for debugging ML-based modeling. Our findings demonstrate that culture-dependent enrichment methods, meteorological variables, and qPCR can be used to identify diverse ARGs associated with clinically relevant Gram-negative bacteria from environmental samples with a low antibiotic resistance profile. Additionally, our study highlights the presence of hospital-contained ARGs, including significant clinically relevant carbapenemase genes, in beach environments as a concerning issue.

To sum up, our study emphasizes the environmental impact of ARGs in beach waters and suggests that monitoring beach waters based on the predictive and explanatory framework of machine learning can be a useful policy direction. The use of ML models can help us gain a better understanding and prediction of the occurrence of ARGs in the environment, which can ultimately aid in the development of more effective strategies to combat antibiotic resistance. Further research in this area is necessary to develop more comprehensive and accurate models that can account for all factors influencing the distribution of ARGs in beach environments.

## Funding

This project was funded by the Deanship of Scientific Research (DSR), 10.13039/501100004054King Abdulaziz University, Jeddah, under grant No. (DF-247-141-1441). The authors, therefore, gratefully acknowledge DSR technical and financial support.

## CRediT authorship contribution statement

**Qandeel Zahra:** Methodology, Visualization, Formal analysis, Writing – review & editing, Formal analysis, Data curation. **Jawaria Gul:** Methodology, Visualization, Formal analysis, Writing – review & editing. **Ali Raza Shah:** Methodology, Visualization, Formal analysis, Writing – review & editing. **Muhammad Yasir:** Conceptualization, Formal analysis, Investigation, Project administration, Resources, Writing – original draft, Funding acquisition. **Asad Mustafa Karim:** Conceptualization, Formal analysis, Investigation, Project administration, Resources, Writing – original draft, Supervision, Formal analysis, Data curation.

## Declaration of Competing Interest

The authors declare that they have no known competing financial interests or personal relationships that could have appeared to influence the work reported in this paper.

## Data Availability

Data will be made available on request.
